# Arterial Complications following Total Knee Arthroplasty (TKA): A Systematic Review and Proposal for Improved Monitoring

**DOI:** 10.5704/MOJ.2303.010

**Published:** 2023-03

**Authors:** H Hodgson, N Saghir, R Saghir, P Coughlin, DJA Scott, A Howard

**Affiliations:** 1 Department of Academic Orthopaedics, Leeds Teaching Hospitals NHS Trust, York, United Kingdom; 2 Department of Plastic Surgery, Wythenshawe Hospital, Manchester, United Kingdom; 3 Department of Orthopaedics, Huddersfield Royal Infirmary, Huddersfield, United Kingdom; 4 Department of Vascular Surgery, Addenbrooke's Hospital, Cambridge, United Kingdom; 5 Leeds Vascular Institute, Leeds General Infirmary, Leeds, United Kingdom; 6 Nuffield Department of Orthopaedics Rheumatology and Musculoskeletal Sciences, University of Oxford, Oxford, United Kingdom

**Keywords:** total knee arthroplasty, complications, vascular, systematic review

## Abstract

**Introduction:**

Total knee arthroplasty (TKA) is a common operation and is becoming more common due to population aging and increasing BMI. TKA provides excellent improvement in quality of life but carries risk of arterial complications in the perioperative period. This systematic review aims to provide a greater understanding of the incidence of such complications, and time taken to diagnose arterial injury.

**Materials and methods:**

PubMed, Medline, Ovid SP and EMBASE databases were searched with the following MeSH keywords: ‘complication’, ‘vascular injury’, ‘ischaemia’, ‘spasm’, ‘thrombosis’, ‘pseudoaneurysm’, ‘transection’, ‘pulse’, ‘ABPI OR ABI’, ‘Doppler’, ‘amputation’. All arterial vascular events in the perioperative state of the total knee replacement were included. Records were independently screened by two reviewers, and data was extracted according to a pre-determined proforma. Overall incidence and time to diagnosis was calculated for complications. Systematic review registration PROSPERO: CRD42018086643. No funding was received.

**Results:**

Twelve studies were selected for inclusion. A total of 3325 cases of arterial complications were recorded across all studies, and were divided into three categories, pseudoaneurysms (0.06%); ischaemia and thrombosis (0.17%); haemorrhage and arterial transections (0.07%). Time taken to reach the diagnosis for each complication was longest in the ischaemia and thrombosis group (6.8 days), followed by pseudoaneurysm (3.5 days) and haemorrhage and transections (3.0 days).

**Conclusion:**

TKA post-operative vascular complications are rare, but when they do occur they lead to limb and life threatening complications. This should be discussed with patients during the consent process. Current times to diagnosis represent missed opportunities to recognise arterial injury and facilitate rapid treatment of the complication. A very low threshold for seeking specialist input should be adopted, and any concern for vascular injury, such as unexplained perioperative bleeding, absent lower limb pulses in the post-operative period or unexplained severe pain should warrant immediate review by a vascular surgeon, and in centres where this is not possible, immediate blue-light transfer to the closest vascular centre.

## Introduction

Total knee arthroplasty (TKA) is a common orthopaedic procedure, replacing the degenerative articulating surfaces of the knee to relieve pain and improve function. The primary indication for TKA is osteoarthritis, accounting for 94-97% of all primary TKAs^1^. Currently there are in excess of 60,000 TKA in England and Wales each year, and in the USA the prevalence of TKA is 1.52%^2^. This number is on the rise due to an ageing population and increasing BMI^3-5^. In addition, knee replacement is increasingly being offered to younger patients as our knowledge and expertise of revision arthroplasty improves^2^. It has been projected that by 2030 the number of TKA operations will increase by 673%6.

Common and well-recognised complications of TKA include joint infection, deep vein thrombosis, and post-operative knee instability^7^. Arterial complications are considered uncommon, and the exact frequencies and clinical sequalae are unclear^8,9^. Vascular injuries are potentially catastrophic, requiring urgent reconstruction with or without fasciotomies, and delays in diagnosis may lead to amputation^8^. Such complications can also have a significant impact on the National Health Service (NHS) and private organisations due to litigation and medico-legal costs^10^. There is a continued focus on the quality of the surgical consent process in clinical practice and ensuring transparency of risks and complications when communicating with patients. A key aspect of consent is to accurately reflect the procedural risks to the patient^11^. The clinical significance of a vascular injury following TKA is substantial, and therefore a contemporary review of the incidence of these injuries and the clinical consequences is urgently required. This systematic review aims to (A) to identify the nature of such vascular injuries; (B) to determine the frequency of vascular complications following knee arthroplasty; (C) to provide a proposal to improve early detection and surveillance of post-operative vascular complications through a simple-to-use observation chart.

## Materials and Methods

A systematic search of the following databases was carried out: Embase Classic, Embase, PubMed, Scopus, and Ovid MEDLINE. The year 1970 was used as the start of the search period to reflect the beginning of routine TKA in clinical practice^12^. The following MeSH terms were used: total knee replacement, total knee arthroplasty, complication; vascular injury; ischaemia OR ischemia; spasm; thrombosis; pseudoaneurysm; transection; pulse; ABPI OR ABI; Doppler; amputation. Reference lists of included papers were searched to ensure all relevant articles were identified. Grey literature was excluded, including unpublished articles, conference proceedings, and case reports. Inclusion and exclusion criteria are shown in (Table I).

**Table I:** Inclusion and exclusion criteria

Category	Included	Excluded
Date	January 1st, 1970 - present day	
Exposure of Interest	All patients included in this study must have undergone a total knee arthroplasty (TKA) and as a result developed an arterial vascular complication within one year post-operatively	
Geographic location of study	All countries will be included	
Language	English	
Participants	Patients of all ages will be included in this study.	
Peer-review	All articles included must be peer-reviewed	Unpublished literature will be excluded
Reported outcomes	Each study must report one or more of the following outcomes to be included amongst the analysis: Vascular assessment performed pre-operatively Vascular assessment performed post-operatively Time takes to diagnose complication Type of vascular complication Imaging modality used to diagnose the complication Treatment of vascular complication	
Setting Study Design	All patients must have received a TKA in a hospital setting Only studies in which 10 or more TKA are to be included	Case reports and case series with less than 10 reported TKA must be excluded
Type of Publication	Only include original published studies	Exclude all reviews, editorials, letters, and case reports

Titles and abstracts were reviewed by two independent reviewers (NS and RS). Records deemed to be potentially relevant were screened by reading the full paper to assess suitability for inclusion. Differences in opinion were settled by consensus. Data extraction was carried out by two independent reviewers according to a pre-determined proforma. The following data were collected from included studies: sample size, mean age, documentation of vascular assessment pre- and post-operatively, type of vascular complication, time to diagnosis, imaging used for diagnosis, treatment of complication, and further complications. For inclusion, studies must have included patients who underwent a TKA, followed up for a minimum of one year, of any age, have been published in a peer-reviewed journal, and reported complication outcomes.

Arterial complications were considered with the following categories respectfully: pseudoaneurysm (false aneurysm formed between two outer layers of an artery), ischaemia and thrombosis (occlusive vessel disease restricting blood flow) and haemorrhage and arterial transections (bleeding and iatrogenic damage to the vessels). Average incidence and time to diagnosis was determined from data provided in articles, and combined values are reported as mean +/standard deviation. The Preferred Reporting Items for Systematic Review and Meta-analysis (PRISMA) guidelines^13^ were used to guide the review process. No funding was received for this study. Systematic review registration PROSPERO: CRD42018086643.

## Results

**Fig. 1:**
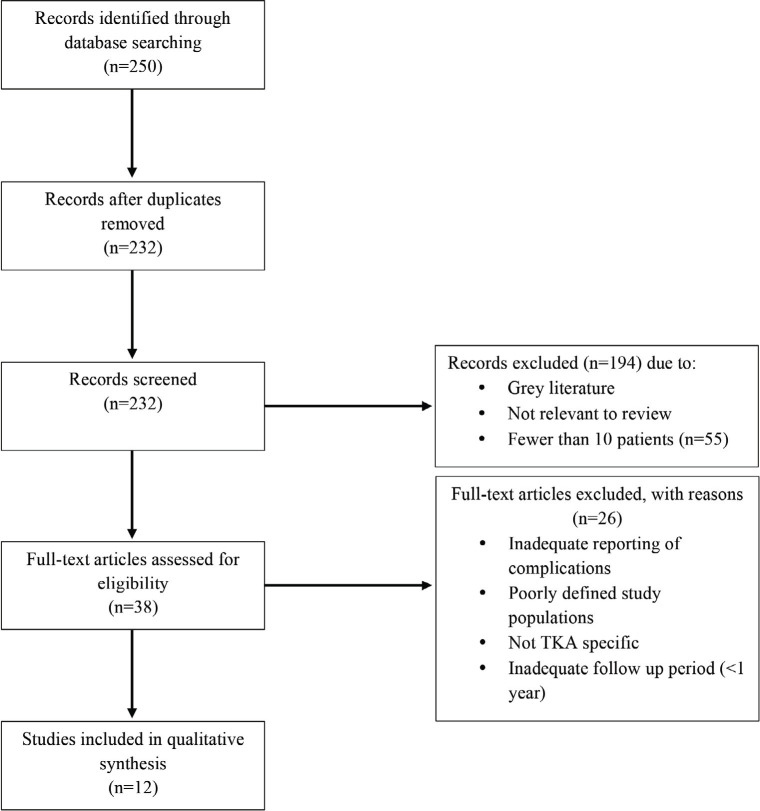
PRISMA flow diagram for study inclusion.

Searches revealed 250 records (Fig. 1). Eighteen duplicate records were excluded, and a further 194 records were excluded. Out of which, 55 had fewer than 10 patients, and the remainder were grey literature or were not relevant to the systematic review. Twenty-six articles were further excluded because they included patients other than TKA, have follow-up of less than one year, or inadequate reporting of complication outcomes. Twelve articles satisfied the inclusion criteria, published between 1992 to 2015 (Table II). Sample sizes for each paper varied between 19 to 3114 cases of arterial complication in the perioperative period following a TKA.

**Table II:** Inclusion and exclusion criteria

Article Name	Year	Author	Number of arterial complications	Type of complication	Time to diagnosis
Popliteal artery pseudoaneurysm after TKA	2016	Ammori *et al*^15^	7 out of 7937	Popliteal artery pseudoaneurysm (n=7)	Mean 15 days (Range 7-27) (n=7)
Acute arterial complications associated with total hip and knee arthroplasty	2003	Calligaro *et al*^17^	24 out of 23199	Acute ischaemia only (n=18) Bleeding only (n=4) Arterial transection (n=5) Arterial pseudoaneurysm (n=5)	>1 day (n=18) 1-5 days (n=14)
The national incidence of iatrogenic popliteal artery injury during total knee replacement	2015	Dua *et al*^18^	43 out of 1297369	Popliteal artery injury (n=43)	>1 day (n=41) 1 day (n=1) 3 days (n=1)
Updated strategies to treat acute arterial complications associated with total knee and hip arthroplasty	2013	Troutman *et al*^19^	37 out of 26374	Ischaemia alone (n=28) Haemorrhage (n=6) Ischaemia with haemorrhage (n=6) Pseudoaneurysm (n=9)	>1 day (n=28) 1-5 days (n=18) 5 to 30 days (n=3)
Vascular complications after total knee arthroplasty – a single institutional experience	2016	Padegimas *et al*^20^	13 out of 9951	Popliteal pseudoaneurysm (n=10) Thromboses (n=3)	>1 day (n=12) 1 day (n=1)
Severe vascular complications and intervention following elective total hip and knee replacement: a 16 years retrospective analysis	2015	Avisar *et al*^21^	3 out of 2073	Haemorrhage (n=1) Arterial thrombosis (n=2)	>1 day (n=1) 10 days (n= 1) 90 days (n=1)
Predictors of lower extremity arterial injury after total knee or total hip arthroplasty	2008	Abularrage *et al*^14^	20 out of 24029	Arterial injury/transection (n=20)	Intra-operatively (n=4) >1 day (n=6) 1-5 days (n=5) 6-30 days (n=5)
Arterial and ischemic aspects of TKA	1992	DeLaurentis *et al*^24^	6 out of 1182	Ischaemia (n=6)	1-7 days (n=6)
Acute arterial thrombosis associated with TKA	1994	Savarese *et al*^16^	7 out of 4097	Arterial thrombosis (n=7)	Not clearly specified
Popliteal artery injury associate with TKA: trends, costs and risk factors	2014	Ko *et al*^25^	3114 out of 5,491,907	Arterial injury (n=3114)	Not clearly specified
Popliteal vascular injury during TKA	2003	Da Silva *et al*^22^	19 cases reported	Arterial injury/transection (n=19)	Not clearly specified
Popliteal artery injury during knee replacement: a population-based nationwide study	2013	Bernhoff *et al*^23^	32 cases reported	Pseudoaneurysms (n=11) Thrombosis (n=7) Arterial injury (n=25)	Not clearly specified

**Fig. 2:**
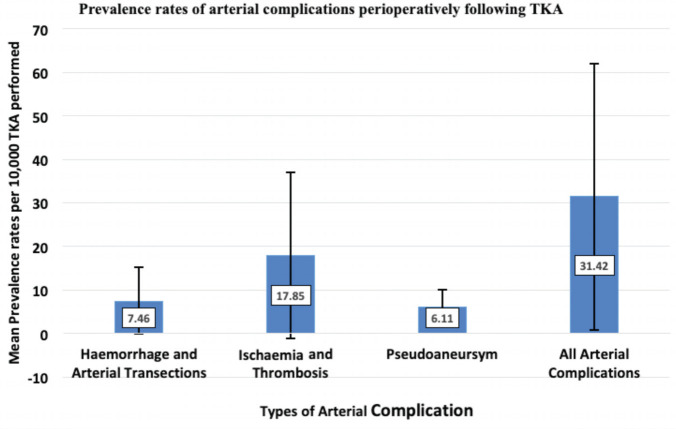
Bar chart with error bars representing the prevalence per 10,000 TKA performed for types of arterial complications developing post-operatively.

**Fig. 3:**
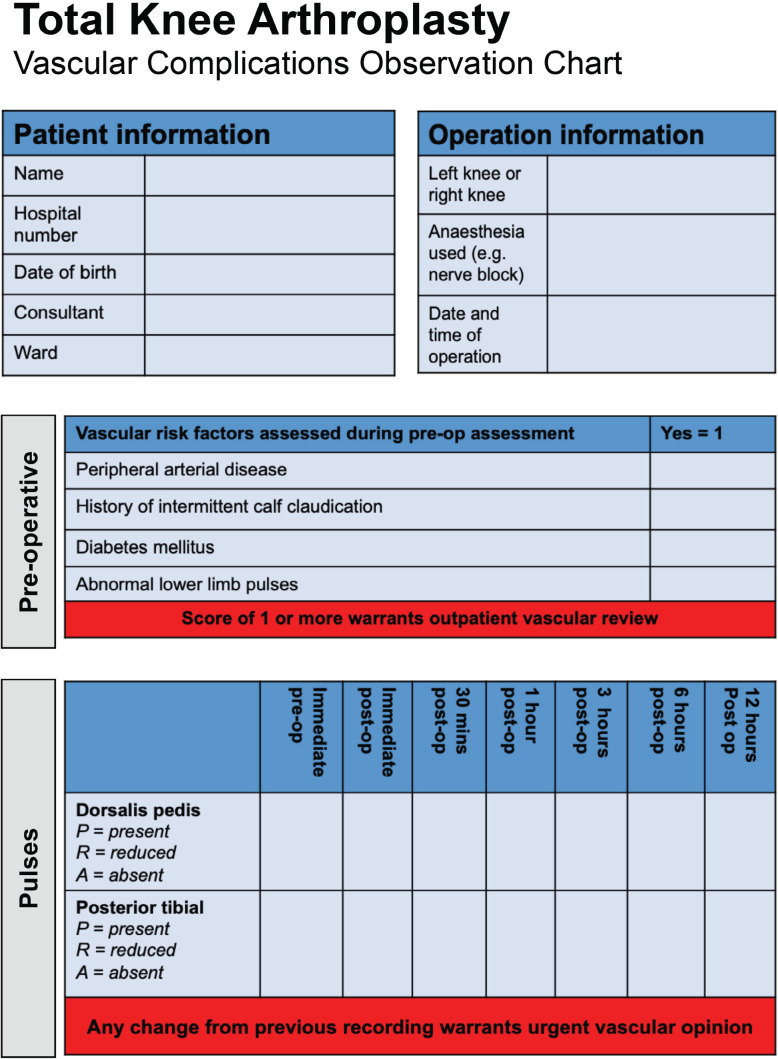

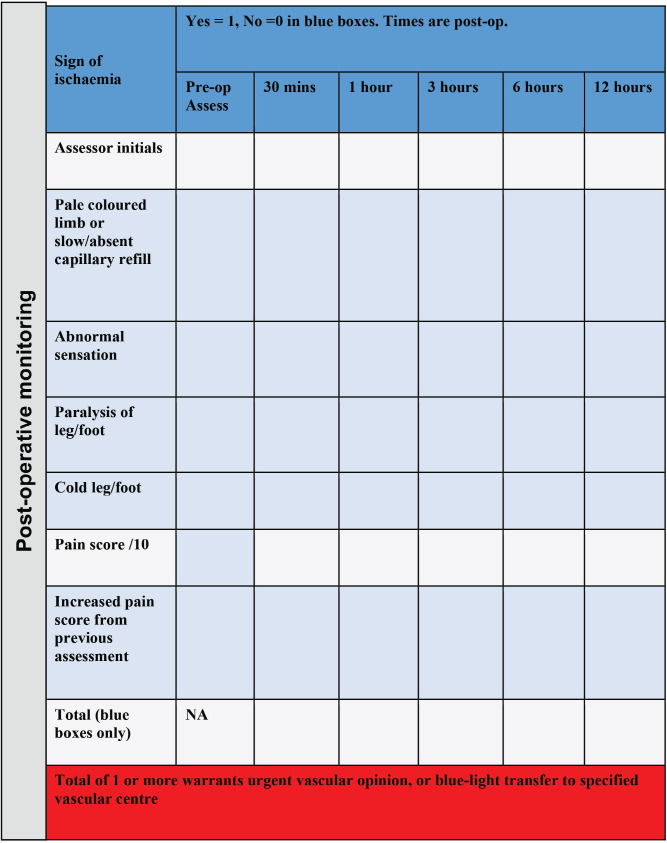
TKA vascular complications observation chart

Prevalence of complications are shown in (Fig. 2). Ischaemia and thrombosis had the highest prevalence rates at 17.85 (±19.09) per 10,000 TKA operations; haemorrhage and arterial transection occurred second most frequently at 7.46 (±7.63) per 10,000 TKAs; least prevalent was pseudoaneurysms at 6.11 (±3.91) per 10,000 TKAs^14-25^. Time taken to diagnose arterial complications perioperatively was 4.81 (±2.25) days; ischaemia and thrombosis took the longest time to diagnose at 6.78 (±1.85) days, followed by pseudoaneurysms 3.49 (±1.42) days then haemorrhage and arterial transections at 3.00 (±5.55) days demonstrated in (Fig. 3)^14-25^.

## Discussion

Total arterial complications following TKA occur in 31.42 (±30.63) per 10,000 TKA or 0.31% of all TKA procedures. Based on the number of TKA in UK each year, 66 patients per annum^3-5^ are at risk of an arterial complication. Whilst it is reassuring that the rate of arterial injury is much lower than other recognised complications of TKA, such as surgical site infection (1.89% to 5.6%), joint stiffness (1.3%) and venous thromboembolic events (2.1%)^26-29^, the consequences of arterial injury have the potential to threaten limb and/or life, and therefore must be discussed with patients during the consent process for TKA.

Following vascular injury, irreversible muscle damage begins after three hours of ischaemia and is complete at six hours^30^. This is a short window where a rapid diagnosis is paramount, and the availability of vascular surgery is essential for providing timely intra-operative resolution of unintended arterial complications^31^. In specialist elective orthopaedic centres without an onsite vascular team, the challenge of getting the patient to the closest vascular centre within a time frame arises. It has been shown that patients who suffer an arterial complication following TKA in a centre without a vascular surgeon onsite have a delay of up to eight hours before repair and are at substantial risk of loss of function^23^. In centres without an onsite vascular team, a standard operating procedure with transfer arrangement to a named vascular centre must be in place when arterial injuries occur.

A further and related complication following orthopaedic interventions is compartment syndrome. The management of this serious complication is aided by the British Orthopaedic Association standardised guideline for compartment syndrome^32^, which must be closely adhered to when this complication is suspected, along with the Royal College of Nursing compartment syndrome specific observation chart^33^.

Current timing to diagnosis of arterial injury represent missed opportunities to recognise arterial injury and facilitate rapid treatment of the complication. It is not immediately clear why some vascular injuries are missed, and is likely due to numerous factors, underpinned by poor awareness of the complications in clinical practice. It is likely that some types of vascular complications are less likely to be apparent in the immediate setting, such as pseudoaneurysm or thrombosis. Additionally, regional anaesthesia may mask or delay the onset of symptoms^34^, and their use should be considered carefully in patients at risk of arterial complications and or compartment syndrome. It is important that clinicians do not use regional blocks to explain symptoms that are concerning for vascular injury. Additionally, perioperative assessment of perfusion may be hampered by current practice in dressing the limb in a compression stocking immediately after closing the wound, and therefore obscuring the operating surgeons assessment of the colour of the limb. It is essential that lower limb pulses are assessed prior to applying the stocking.

Within the orthopaedic discipline, the incidence of litigation is rapidly rising^35^. For knee replacement surgery, vascular injuries represent 4.2% of claims, and are the most expensive cause of claims with a mean cost per claim of £232,90036, representing a significant annual cost to the NHS. Additionally, failure to warn patients about inherent risks represents a substantial proportion of claims^37^, and may represent the most straightforward method of reducing them. An assessment by the operating surgeon of lower limb pulses should take place in the pre-operative and immediate postoperative setting for any patient undergoing TKA to aid surveillance of arterial injury requiring further management. A very low threshold for seeking specialist input should be adopted, and any concern for vascular injury, such as unexplained perioperative bleeding, absent lower limb pulses in the post-operative period or unexplained severe pain should warrant immediate review by a vascular surgeon, and in centres where this is not possible, immediate blue-light transfer should be activated to the closest vascular centre.

A simple observation chart (Fig. 3) for active surveillance of post-operative vascular complications may reduce time to diagnosis and improve clinical awareness but requires validation in an orthopaedic setting. Ideally, post-operative patients should have regular post-operative assessment of lower limb pulses by specialist vascular nursing staff. When this is not possible, novel methods of assessing peripheral vascular status, such as devices which measure systolic toe pressure, may be simple and useful for diagnosing vascular injury in future practice^38^ and their validity should also be assessed in future research. Finally, patients at risk should be identified during routine pre-operative screening, and patients with a history of diabetes or peripheral arterial disease (or any concerns for undiagnosed peripheral arterial disease including intermittent calf claudication) should be referred to the vascular team for further review prior to surgery. Further research should be carried out to help validate this observation chart in its ability to identify arterial complications in a timely and thorough fashion. The chart will also benefit from further development in collaboration with the British Orthopaedic Association, Vascular Society and Royal College of Nursing.

The topic of vascular injury following TKA has not been widely studied, and only 12 studies were suitable for inclusion. There was great variability in the reported information, likely due to differing outcomes, definitions, reporting methods and sample sizes. In part, this was due to inclusion of studies from research groups across the world, resulting in varying surgical approaches, surgical standards and types of prosthetics.

It was not possible to comment on the long term sequalae of arterial complications due to the lack of data in the literature on the topic. The lack of information in the previously published studies also hinders the ability to perform a combined analysis, or a meaningful sub-group analysis to determine at risk groups for arterial complications. Furthermore, we were unable to analyse the post-operative recovery and in particular the vascular status of the patients pre- and post-operatively as this was poorly documented by studies. This raises the question over whether the preoperative vascular status of the limb should be documented prior to surgery including the presence of a past medical history of intermittent claudication, palpation of the lower limb pulses and the measurement of the ankle brachial pressure index. We therefore suggest better and more thorough reporting of such information in future publications.

## Conclusion

This systematic review raises awareness of operating surgeons to arterial complications associated with primary TKA. Our review allows a consenting surgeon to quote an arterial complication rate of 0.3%. However, in those patients who have a popliteal artery transection and a subsequent vascular reconstruction there are long term sequalae of fasciotomy wounds, nerve injuries including foot drop and neuropathic pain, and the potential for prosthetic infections, all of which increase the risk of major amputation. We highlight that there is currently a delay in diagnosing arterial complications and suggest using a simple observation chart for monitoring post-operative vascular complications to facilitate earlier identification. A vascular team onsite or standard operating procedures which enable rapid transfer to a specialist vascular centre may also improve outcomes.
